# Impacts of lockdown on dietary patterns among youths in China: the COVID-19 Impact on Lifestyle Change Survey

**DOI:** 10.1017/S1368980020005170

**Published:** 2021-05-17

**Authors:** Bin Yu, Dong Zhang, Wanqi Yu, Miyang Luo, Shujuan Yang, Peng Jia

**Affiliations:** 1 West China Second Hospital, Sichuan University, Chengdu, China; 2 Department of Family and Preventive Medicine, University of Arkansas for Medical Sciences, Little Rock, AR, USA; 3 West China School of Public Health and West China Fourth Hospital, Sichuan University, Chengdu, China; 4 Xiangya School of Public Health, Central South University, Changsha, China; 5 Department of Land Surveying and Geo-Informatics, The Hong Kong Polytechnic University, Hong Kong, China; 6 International Institute of Spatial Lifecourse Epidemiology (ISLE), Hong Kong, China

**Keywords:** COVID-19, Youth, Lockdown, Diet, Eating

## Abstract

**Objective:**

To assess changes in dietary patterns among youths in China after COVID-19 lockdown.

**Design:**

This study was based on the COVID-19 Impact on Lifestyle Change Survey (COINLICS), a national retrospective survey established in early May 2020. The questionnaire was distributed through social media platforms. The sociodemographic information and routine dietary patterns before and after lockdown of participants were investigated. *t* tests and *χ* ^2^ tests were used to compare the differences in consumption patterns of twelve major food groups and beverages between sex and across educational levels before and after lockdown. Factor analysis was employed to obtain the main dietary patterns.

**Settings:**

China.

**Participants:**

A total of 10 082 youths.

**Results:**

A significant decrease was observed in the average weekly frequency of rice intake, while significant increases were observed in the frequency of intake of wheat products, other staple foods, fish, eggs, fresh vegetables, preserved vegetables, fresh fruit and dairy products (all *P* values < 0·01). Heterogeneities of average weekly frequency existed between sex and across educational levels to different extents. The three main dietary patterns derived were loaded most heavily on dairy products, rice and wheat products, separately; the rice pattern became more dominant than the wheat products pattern after lockdown. The frequency of sugar-sweetened beverage consumption had decreased, while the frequency of other beverages had increased.

**Conclusions:**

Our timely survey would inform policymakers and health professionals of these significant changes in youths’ dietary patterns after lockdown, with heterogeneities observed to different extents between sex and across educational levels, for better policy-making and public health practice.

The world has been dramatically changing since early 2020 due to the pandemic caused by the novel coronavirus, also known as COVID-19^(1)^. To curb the spread of the COVID-19, many countries and regions have adopted serious measures to maintain social distancing, such as the stay-at-home decree and lockdowns^(2)^. In the fear of food shortage during the COVID-19 pandemic and the accompanying lockdown measures, many people around the globe stocked up on food to prepare for the unpredictable pandemic, which may lead to significant changes in lifestyle and dietary patterns^(3)^. For instance, COVID-19 confinement in Spain has led to decreased intake of fried foods, snacks and fast foods, but increased intake of the Mediterranean diet-related foods^(4)^. Also, in Italy, the consumption of home-made desserts, bread and pizza has increased, while the consumption of savoury snacks and processed meat has decreased^(5)^. In China, most food outlets (e.g. food markets and restaurants) were closed during the lockdown, with a limited number of open outlets accessible to only those living within the communities. The variety and supply of food in the outlets also differed across communities. Stocking up on food was inevitable, with changes in dietary patterns during lockdown observed^(6)^.

The youth population has been proven vulnerable to the changes in lifestyle and dietary patterns compared with the general adults^(7)^, which might be also observed during the COVID-19 pandemic. Currently, although the lockdown was lifted in China, maintaining social distance, minimising outside activities and reducing population mobility were still recommended by official institutions. Until May, some schools in China (especially in high-risk provinces like Hubei, Heilongjiang) remain closed and even required students not to return the semester for fear of a second-wave outbreak. These situations forced many students to stay at home for long periods of time. However, as a previous study found, youths will fare worse on weight control lifestyle while at home compared to when they are engaged in their usual school curriculum^(8)^. Specifically, youths might be inclined to develop and stick to a sedentary lifestyle from heavy screen use (watching TV, searching the internet or taking online courses) and no space for physical activities. Under poor conscious of self-health management, this lifestyle is associated with ‘mindless’ eating and may lead them to suffer dietary patterns changes (e.g. consumption of high-energy foods more frequently)^(9,10)^. In addition, adverse psychological conditions related to the epidemic, as well as the persistent psychological trauma, also affect their dietary patterns. For example, stress from unpredictable pandemic may lead subjects towards overeating, especially ‘comfort foods’ rich in sugar, defined as ‘food craving’, which is associated with high carbohydrates intake^(5,11)^.

What is more serious and worthy of further attention is that the lockdown lifted might exist for a long time, especially the vaccine has not been developed while another outbreak is expected this winter^(12)^. As a result, the changed dietary patterns and its accompanying health effects on youths may be lasting. Although in early April, closed to the end of the full lockdown, authorities and official organisations in China had recommended some nutritional dietary guidelines^(13)^, the youths’ daily dietary patterns and lifestyle formed during long-term lockdown might be not easy to be changed or corrected. Since youths’ dietary patterns have been proved to be closely related to some chronic diseases like obesity and diabetes in later life^(14)^, the potential changes in dietary patterns among this vulnerable population are of concern.

This study aimed to provide a picture of changes in dietary patterns of the Chinese youths after the COVID-19 lockdown was lifted. Our findings would provide empirical evidence for the following interventions and policies to counteract negative impact on dietary patterns that may last long. They may also serve as a reference for other countries to raise awareness of or developing solutions to this issue.

## Methods

### Study design and data collection

A national retrospective survey called the COVID-19 Impact on Lifestyle Change Survey (COINLICS) was carried out in early May 2020 in China^(15)^. Youth participants under three educational attachments (i.e. high school, college or graduate) in China was recruited using a snowball sampling method. A web-based questionnaire was distributed among several social media groups of educators at all three levels formed during the national conferences in the field. In each province of China, at least two educators shared the questionnaire with their eligible students around through chat groups and/or two mainstream social media platforms (i.e. WeChat and Tencent QQ). Those students who had finished the questionnaire were encouraged to forward it to others.

All recruited participants have voluntarily reported their basic sociodemographic information and routine lifestyles in the month immediately before the COVID-19 lockdown (January 2020, hereafter referred to as *before lockdown*) and after the lockdown was lifted (May 2020, hereafter referred to as *after lockdown*). An informed consent notice was highlighted on the front page of the questionnaire, and only those who agreed to participate and clicked the ‘agree’ button were allowed to continue with the questionnaire. Three commonsensical questions (e.g. where the capital of China is) were placed among the questions to test the validity of the questionnaire. If any of them were answered incorrectly, that questionnaire was considered invalid. The questionnaire was designed to be completed online anonymously. A total of 10 082 individuals from 31 provinces/municipalities participated in the study.

### Measures of dietary patterns

Dietary patterns in the study referred to two parts, consisting of food consumption and beverage consumption. A total of 12 major food groups consumed by the Chinese citizens were investigated, including rice, wheat products, other staple foods, meat, poultry, fish, eggs, dairy products, fresh vegetables, preserved vegetables, fresh fruit and soyabean products^(16)^. Participants were asked to recall and answer the species and frequencies of their habitual food consumption before and after lockdown, which were further grouped to obtain the main dietary patterns. Besides, participants were requested to answer the types of beverages mainly consumed in the two periods, including sugar-sweetened beverages (SSB), coffee and caffeinated drinks, tea, and other beverages (e.g. fruit juice). Four categories of frequency of consumption (‘daily’, ‘4–6 d/week’, ‘1–3 d/week’ and ‘none’) were listed and all participants chose the most suitable answer based on their habitual food and beverage consumption.

### Statistical analyses

Descriptive statistics were used to summarise the characteristics of participants by expressing the results as mean (sd) for continuous variables that follow the normal distribution, median (p25, p75) for continuous variables that do not follow the normal distribution, or percentages for categorical variables. All the categories of frequency related to food and beverage consumption were converted into continuous variables (i.e. ‘daily’ was denoted by 7 d/week, ‘4–6 d/week’ by 5 d/week, ‘1–3 d/week’ by 2 d/week and ‘none’ by 0) to enable an intuitive comparison of dietary intakes in the two periods. Paired or independent *t* tests for continuous variables and *χ* ^2^ tests for categorical variables were used to compare the differences in frequency of food and beverage consumption between sex and across educational levels before and after lockdown. For the twelve groups of food, factor analysis with orthogonal rotation was employed to derive the main dietary patterns before and after lockdown. The number of factors retained was based on eigenvalues > 1·0 and the breakpoint identified in the scree plot^(17)^. Factor loading of > |0·20| was included to represent the food strongly associated with the identified factor^(18)^. R (R Core Team, 2020) was used for statistical analyses^(19)^.

## Results

A total of 10 082 eligible youths was included in this study. The average age of the participants was 19·8 ± 2·3 years old (Table 1). The majority of them were female (71·7 %), Han nationality (95·3 %), non-urban residence (63·2 %) and from the western region (87·9 %). The most common annual household income category is 12 000–20 000 RMB (28·7 %), and the most common major among included students is social science (38·2 %). None of the high school students from the northeastern and central regions participated in the survey. There were no significant differences in the age and ethnic distribution between males and females. There remain significant changes of youths’ weekly frequency of major food intake after lockdown, with differences between sex and across educational levels in some dietary types (see online supplementary material, Supplemental Table S1).

**Table 1:** Basic characteristics of the participating youths*

	High school students (%)			Undergraduate students (%)			Graduate students (%)			All (%)
Variables	Male (*n* 678)	Female (*n* 2146)	Total (*n* 2824)	Male (*n* 2106)	Female (*n* 4918)	Total (*n* 7024)	Male (*n* 68)	Female (*n* 166)	Total (*n* 234)	Grand total (*n* 10 082)
Age (year)										
Mean	17·5^a^	17·5^a^	17·5^a^	20·6^a^	20·6^a^	20·6^a^	24·3^a^	24·7^a^	24·6^a^	19·8
sd	1·2	1·2	1·2	2·0	1·6	1·8	4·1	3·2	3·5	2·3
Ethnic										
Han	96·9^a^	96·7^a^	96·7^a^	94·2^a^	95·1^a^	94·8^a^	92·6^a^	92·2^a^	92·3^a^	95·3
Minority	3·1^a^	3·3^a^	3·3^a^	5·8^a^	4·9^a^	5·2^a^	7·4^a^	7·8^a^	7·7^a^	4·7
Urbanicity										
Urban	31·9^a,b^	22·1^a,b^	24·4^a^	40·2^a^	41·3^a^	41·0^a^	57·4^a^	63·3^a^	61·5^a^	36·8
Non-urban	68·1^a,b^	77·9^a,b^	75·6^a^	59·8^a^	58·7^a^	59·0^a^	42·6^a^	36·7^a^	38·5^a^	63
Region†										
Northeast	–	–	–	0·3^a,b^	0·2^a,b^	0·3^a^	2·9^a^	4·2^a^	3·8^a^	0·3
East	0·6^a^	0·7^a^	0·7^a^	10·1^a,b^	12·2^a,b^	11·5^a^	25·0^a^	28·3^a^	27·4^a^	8·9
West	99·4^a^	99·3^a^	99·3^a^	85·5^a,b^	84·1^a,b^	84·5^a^	61·8^a^	50·0^a^	53·4^a^	87·9
Central	–	–	–	4·1^a,b^	3·5^a,b^	3·7^a^	10·3^a^	17·5^a^	15·4^a^	2·9
Household income (*yuan*/year)										
< 12 000	20·8^a,b^	25·7^a,b^	24·5^a^	21·7^a^	18·5^a^	19·4^a^	13·2^a^	4·3^a^	6·9^a^	20·6
≥ 12 000–20 000	28·0^a,b^	37·9^a,b^	35·5^a^	23·2^a^	28·2^a^	26·7^a^	4·5^a^	11·4^a^	9·4^a^	28·7
≥ 20 000–60 000	28·3^a,b^	25·0^a,b^	25·8^a^	26·3^a^	28·1^a^	27·6^a^	14·7^a^	24·7^a^	21·8^a^	27·0
≥ 60 000–100 000	15·2^a,b^	7·8^a,b^	9·6^a^	14·8^a^	13·4^a^	13·8^a^	22·1^a^	22·9^a^	22·6^a^	12·8
≥ 100 000–200 000	5·5^a,b^	2·6^a,b^	3·3^a^	9·4^a^	8·6^a^	8·9^a^	27·9^a^	24·7^a^	25·6^a^	7·7
≥ 200 000	2·2^a,b^	1·0^a,b^	1·3^a^	4·6^a^	3·2^a^	3·6^a^	17·6^a^	12·0^a^	13·7^a^	3·2
Major										
Medical Science	59·0^a,b^	98·4^a,b^	89·0^a^	12·4^a,b^	16·0^a,b^	14·9^a^	41·2^a,b^	52·4^a,b^	49·1^a^	36·4
Science/Engineering	39·4^a,b^	1·0^a,b^	10·2^a^	51·9^a,b^	22·6^a,b^	31·4^a^	44·1^a,b^	20·5^a,b^	27·4^a^	25·4
Social Science	1·6^a,b^	0·6^a,b^	0·8^a^	35·7^a,b^	61·4^a,b^	53·7^a^	14·7^a,b^	27·1^a,b^	23·5^a^	38·2

* Values under a given variable were marked with the superscript a, if the difference across educational levels (high school students, undergraduate students and graduate students) within the overall population (total) or within a given sex (male, female) was significant (*P* < 0·05); and marked with the superscript b, if the difference between sex within a given educational level was significant (*P* < 0·05).

† Northeast (Liaoning, Jilin and Heilongjiang), East (Beijing, Tianjin, Hebei, Shanghai, Jiangsu, Zhejiang, Fujian, Shandong, Guangdong and Hainan), Central (Shanxi, Anhui, Jiangxi, Henan, Hubei and Hunan) and West of China (Inner Mongolia, Guangxi, Chongqing, Sichuan, Guizhou, Yunnan, Tibet, Shaanxi, Gansu, Qinghai, Ningxia and Xinjiang).

We observed a significant decrease in the average weekly frequency of rice consumption, while significant increases in other staple foods, fish, eggs, fresh vegetables, preserved vegetables, fresh fruit and dairy products after lockdown (all *P* values < 0·05) (Table 2). Meanwhile, differences remained in the average weekly frequency of food intake for students with different sex or educational levels. Specifically, female high school students consumed rice less frequently, while consuming other staple foods, eggs and preserved vegetables more frequently; male undergraduate students consumed rice less frequently, while consuming eggs, preserved vegetable and fresh fruit more frequently after lockdown (all *P* values < 0·05). Also, at the same time, there were remain differences in the average weekly frequency of food intake between sex, with males eating wheat products, other staple foods, meat, poultry, fish, eggs, preserved vegetables, soyabean products and dairy products more frequently, while females eating rice, fresh vegetables and fresh fruit more frequently. Compared to other educational levels, high school students consumed rice more frequently; undergraduate students consumed persevered vegetables more frequently; graduate students consumed most food more frequently except rice and preserved vegetables.

**Table 2:** The average weekly frequency of major food intake among participating youths before and after lockdown

	High school students						Undergraduate students						Graduate students						All	
	Male (*n* 678)		Female (*n* 2146)		Total (*n* 2824)		Male (*n* 2106)		Female (*n* 4918)		Total (*n* 7024)		Male (*n* 68)		Female (*n* 166)		Total (*n* 234)		Grand total (*n* 10 082)	
Variables	Mean	SD	Mean	SD	Mean	SD	Mean	SD	Mean	SD	Mean	SD	Mean	SD	Mean	SD	Mean	SD	Mean	SD
Rice																				
Pre-lockdown	6·3	2·0^a,b^	6·5	1·6^a,b^	6·4	1·7^a^	5·7	2·5^a,b^	5·8	2·2^a,b^	5·8	2·3^a^	5·1	2·7^a^	5·4	2·4^a^	5·3	2·5^a^	5·9	2·2
Post-lockdown	6·3	2·0^a^	6·4	1·8^a,^***	6·4	1·8^a,^***	5·6	2·5^a,^***	5·7	2·4^a,^***	5·6	2·4^a,^***	4·9	2·8^a^	4·8	2·8^a,^***	4·8	2·8^a,^***	5·8	2·3***
Wheat products																				
Pre-lockdown	3·2	2·5^b^	2·8	2·2^b^	2·9	2·3	3·2	2·5^b^	2·8	2·3^b^	2·9	2·3	3·0	2·7	2·4	2·1	2·6	2·3	2·9	2·3
Post-lockdown	3·3	2·6^b^	2·8	2·3^b^	2·9	2·4	3·3	2·5^b,^**	2·9	2·4^b,^**	3·0	2·4***	3·2	2·9	2·8	2·5**	2·9	2·6**	3·0	2·4***
Other staple foods																				
Pre-lockdown	2·0	2·4^b^	1·5	2·1^a,b^	1·6	2·2^a^	2·1	2·5^b^	1·7	2·1^a,b^	1·8	2·2^a^	1·9	2·4	1·8	2·1^a^	1·8	2·1^a^	1·7	2·2
Post-lockdown	2·1	2·4^b^	1·6	2·1^a,b,^***	1·7	2·2^a,^***	2·1	2·5^b,^**	1·8	2·2^a,b,^***	1·9	2·3^a,^***	1·9	2·3	2·0	2·3^a^	2·0	2·3^a^	1·9	2·3***
Meat																				
Pre-lockdown	4·0	2·5^b^	3·2	2·3^a,b^	3·4	2·4^a^	4·1	2·5^b^	3·7	2·4^a,b^	3·8	2·5^a^	4·1	2·7	4·3	2·6^a^	4·2	2·6^a^	3·7	2·5
Post-lockdown	4·0	2·5^b^	3·1	2·3^a,b,^**	3·3	2·4^a,^**	4·1	2·6^b^	3·7	2·5^a,b^	3·8	2·5^a^	4·0	2·7	4·2	2·6^a^	4·1	2·6^a^	3·7	2·5**
Poultry																				
Pre-lockdown	2·0	1·8^a,b^	1·5	1·6^a,b^	1·6	1·7^a^	2·2	2·0^a,b^	1·8	1·7^a,b^	1·9	1·8^a^	2·7	2·1^a^	2·4	2·0^a^	2·5	2·0^a^	1·9	1·8
Post-lockdown	2·0	1·9^a,b^	1·5	1·6^a,b,^*	1·6	1·7^a^	2·2	2·1^a,b^	1·8	1·8^a,b^	1·9	1·9^a^	2·4	2·3^a^	2·5	1·9^a^	2·4	2·0^a^	1·9	1·8
Fish																				
Pre-lockdown	0·9	1·5^a,b^	0·5	1·1^a,b^	0·6	1·3^a^	1·1	1·7^a,b^	0·8	1·3^a,b^	0·9	1·5^a^	1·3	1·4^a^	1·3	1·6^a^	1·3	1·6^a^	0·8	1·4
Post-lockdown	0·9	1·6^a,b^	0·6	1·2^a,b,^*	0·7	1·3^a^	1·2	1·8^a,b,^*	0·9	1·4^a,b,^***	1·0	1·5^a,^***	1·4	1·6^a^	1·4	1·7^a^	1·4	1·6^a,^*	0·9	1·5***
Eggs																				
Pre-lockdown	2·8	2·2^a,b^	2·3	2·0^a,b^	2·4	2·0^a^	3·3	2·4^a,b^	3·1	2·4^a,b^	3·1	2·4^a^	3·8	2·5^a^	4·1	2·6^a^	4·0	2·6^a^	3·0	2·3
Post-lockdown	2·9	2·3^a,b^	2·4	2·0^a,b,^***	2·5	2·1^a,^***	3·4	2·5^a,^***	3·3	2·4^a,^***	3·3	2·4^a,^***	3·9	2·6^a^	4·5	2·6^a,^***	4·3	2·6^a,^***	3·1	2·4***
Fresh vegetables																				
Pre-lockdown	5·1	2·3^b^	5·6	2·2^a,b^	5·4	2·2^a^	5·0	2·4^b^	5·4	2·2^a,b^	5·3	2·3^a^	5·2	2·3	5·8	2·1^a^	5·6	2·2^a^	5·3	2·3
Post-lockdown	5·1	2·4^b^	5·6	2·2^a,b,^*	5·5	2·2	5·1	2·4^b,^*	5·5	2·1^a,b,^***	5·4	2·2***	4·9	2·5^b^	5·9	2·0^a,b^	5·6	2·2	5·4	2·2***
Preserved vegetables																				
Pre-lockdown	1·8	2·0^b^	1·2	1·7^a,b^	1·3	1·8^a^	1·8	2·1^b^	1·3	1·8^a,b^	1·4	1·9^a^	1·2	2·0	1·1	1·8^a^	1·1	1·8^a^	1·4	1·9
Post-lockdown	1·8	2·0^a,b^	1·3	1·7^a,b,^***	1·4	1·8^a,^**	1·9	2·2^a,b,^***	1·4	1·9^a,b,^***	1·6	2·0^a,^***	1·3	2·0^a^	1·4	2·0^a,^*	1·3	2·0^a,^*	1·5	2·0***
Fresh fruit																				
Pre-lockdown	3·4	2·4	3·6	2·3^a^	3·5	2·3^a^	3·4	2·4^b^	3·9	2·4^a,b^	3·7	2·4^a^	3·6	2·4^b^	4·7	2·4^b^	4·4	2·4^a^	3·7	2·4
Post-lockdown	3·4	2·4	3·6	2·4^a^	3·5	2·4^a^	3·5	2·4^b,^***	4·1	2·4^a,b,^***	3·9	2·4^a^***	3·7	2·4^b^	4·9	2·4^b^	4·5	2·4^a^	3·8	2·4***
Soyabean products																				
Pre-lockdown	1·8	2·1^a,b^	1·3	1·8^a,b^	1·4	1·9^a^	2·0	2·1^a,b^	1·8	2·0^a,b^	1·9	2·1^a^	2·4	2·3^a^	2·4	2·3^a^	2·4	2·3^a^	1·7	2·0
Post-lockdown	1·7	2·1^a,b,^**	1·2	1·7^a,b,^**	1·3	1·8^a,^***	2·0	2·2^a,b^	1·7	2·0^a,b,^***	1·8	2·1^a,^***	2·4	2·4^a^	2·5	2·3^a^	2·5	2·4^a^	1·7	2·0***
Dairy products																				
Pre-lockdown	3·0	2·5^b^	2·2	2·1^a,b^	2·4	2·3^a^	3·2	2·5^b^	3·0	2·4^a,b^	3·1	2·5^a^	3·4	2·7	3·3	2·6^a^	3·4	2·6^a^	2·9	2·4
Post-lockdown	2·9	2·5^b^	2·3	2·2^a,b^	2·4	2·3^a^	3·2	2·5^b,^*	3·1	2·5^a,b,^***	3·2	2·5^a,^***	3·5	2·7	3·4	2·6^a^	3·4	2·6^a^	3·0	2·5***

Values under a given variable were marked with the superscript a, if the difference across educational levels (high school students, undergraduate students and graduate students) within the overall population (total) or within a given sex (male, female) was significant (*P* < 0·05); marked with the superscript b, if the difference between sex within a given educational level was significant (*P* < 0·05); and marked by asterisks, if the difference before and after lockdown within a given educational level and sex was significant (*P < 0·05, **P < 0·01, ***P < 0·001).

The three main dietary patterns were determined before and after lockdown: Pattern A was loaded heavily on dairy products, soyabean products and fresh fruit; Pattern B was loaded heavily on rice, fresh vegetables and meat; Pattern C was loaded heavily on wheat products, other staple foods and preserved vegetables (Table 3). Together, the three patterns explain 51·0 % (pre-lockdown) and 51·3 % (post-lockdown) of the variance in dietary patterns. After lockdown, although the food items under these dietary patterns did not change, loading changes of certain foods were observed. Notably, Pattern C was replaced by Pattern B as the second main dietary pattern after lockdown, indicating more frequent consumption of wheat products and preserved vegetables while less frequent consumption of rice, fresh vegetables and meat.

**Table 3:** Factor loadings of the food groups for the main dietary patterns identified among the participating youths before and after lockdown

	Pre-lockdown			Post-lockdown		
Food items	Pattern A	Pattern B	Pattern C	Pattern A	Pattern C	Pattern B
Rice	−0·165	0·750*	0·128	−0·122	0·105	0·771*
Wheat products	−0·060	0·262*	0·769*	−0·092	0·743*	0·296*
Other staple foods	0·236*	0·037	0·722*	0·204*	0·703*	0·034
Meat	0·296*	0·595*	0·203*	0·350*	0·223*	0·561*
Poultry	0·542*	0·196	0·248*	0·561*	0·289*	0·141
Fish	0·567*	−0·120	0·327*	0·551*	0·355*	−0·147
Eggs	0·592*	0·335*	0·079	0·621*	0·094	0·260*
Fresh vegetables	0·324*	0·696*	−0·095	0·403*	−0·109	0·659*
Preserved vegetables	0·363*	−0·058	0·453*	0·253*	0·510*	−0·030
Fresh fruit	0·641*	0·330*	−0·053	0·681*	−0·027	0·277*
Soyabean products	0·646*	−0·111	0·265*	0·600*	0·371*	−0·168
Dairy products	0·712*	0·141	0·023	0·724*	0·073	0·08
**Indices**						
Eigenvalues	2·711	1·781	1·632	2·764	1·741	1·653
Proportion of variation	22·593	14·843	13·600	23·042	14·511	13·782
Cumulative proportion	22·593	37·436	51·036	23·042	37·553	51·335

* Coefficients of > |0·20|. Pattern A: loaded most heavily on dairy products; Pattern B: loaded most heavily on rice; Pattern C: loaded most heavily on wheat products.

Totally, the frequency of all types of beverage consumption had decreased (Table 4). We also observed significant decreases in the average weekly frequency of beverage consumption among female high school students, male and female undergraduate students, and male graduate students (all *P* values < 0·05). In terms of the types of beverage, a majority of the participants consumed SSB in both periods, although the percentage had reduced slightly after lockdown (78·7 to 75·3 %, *P* < 0·001). NS, but a decreased SSB consumption was found among female graduate students (79·1 to 78·0 %). However, by individual level, a larger percentage of increased SSB consumption was observed among female graduate students (9·1 %) than any other group. In contrast, the percentage of participants consuming other beverages had increased (18·1 to 20·3 %, *P* < 0·05) (Table 4).

**Table 4:** Patterns of beverage consumption among participating youths before and after lockdown

	High school students (%)			Undergraduate students (%)			Graduate students (%)			All (%)
Variables	Male (*n* 678)	Female (*n* 2146)	Total (*n* 2824)	Male (*n* 2106)	Female (*n* 4918)	Total (*n* 7024)	Male (*n* 68)	Female (*n* 166)	Total (*n* 234)	Grand total (*n* 10 082)
All types of drinks										
Frequency of consumption (days/week)										
Pre-lockdown										
None	60·3^a,b^	71·9^a,b^	69·1^a^	54·9^a,b^	63·9^a,b^	61·2^a^	63·2^a^	74·1^a^	70·9^a^	63·6
1–3	34·2^a,b^	24·7^a,b^	27·0^a^	37·9^a,b^	31·7^a,b^	33·6^a^	26·5^a^	19·9^a^	21·8^a^	31·5
4–6	2·4^a,b^	1·6^a,b^	1·8^a^	3·4^a,b^	2·0^a,b^	2·4^a^	4·4^a^	1·8^a^	2·6^a^	2·2
7	3·1^a,b^	1·8^a,b^	2·1^a^	3·8^a,b^	2·4^a,b^	2·8^a^	5·9^a^	4·2^a^	4·7^a^	2·7
Post-lockdown										
None	63·8^b^	76·8^a,b,^***	73·7^a,^***	62·6^b,^***	70·7^a,b,^***	68·3^a,^***	66·2	75·9^a^	73·1^a^	69·9***
1–3	30·7^b^	20·1^a,b,^***	22·7^a,^***	30·0^b,^***	25·0^a,b,^***	26·5^a,^***	25·0	19·3^a^	20·9^a^	25·3***
4–6	2·8^b^	1·7^a,b,^***	1·9^a,^***	3·6^b,^***	2·2^a,b,^***	2·6^a,^***	5·9	1·2^a^	2·6^a^	2·4***
7	2·7^b^	1·4^a,b,^***	1·7^a,^***	3·8^b,^***	2·1^a,b,^***	2·6^a,^***	2·9	3·6^a^	3·4^a^	2·4***
Averaged frequency of consumption (days/week)										
Pre-lockdown										
Mean	1·0	0·7	0·8	1·2	0·9	1·0	1·2	0·8	0·9	0·9
sd	1·6^a,b^	1·3^a,b^	1·4^a^	1·7^a,b^	1·5^a,b^	1·5^a^	1·9^a,b^	1·6^a,b^	1·7^a^	1·5
Post-lockdown										
Mean	0·9	0·6	0·7	1·0	0·8	0·8	1.0	0·7	0·8	0·9
sd	1·5^b^	1·3^a,b,^***	1·3^a,^***	1·7^b,^***	1·4^a,b,^***	1·5^a,^***	1.7^b,^*	1·5^a,b^	1·6^a^	1·6***
Type of drinks										
Sugar-sweetened beverages										
Pre-lockdown	67·7^b^	76·3^a,b^	73·6^a^	74·5^b^	83·4^a,b^	80·3^a^	76·0	79·1^a^	77·9^a^	78·7
Post-lockdown	63·4^a,b^	73·2^a,b^	70·0^a^	72·9^a,b^	79·4^a,b,^**	77·1^a,^**	73·9^a^	78·0^a^	76·6^a^	75·3**
Coffee and caffeinated drinks										
Pre-lockdown	6·3	3·8^a^	4·6^a^	10·2^b^	7·9^a,b^	8·7^a^	12·0	14·0^a^	13·2^a^	7·8
Post-lockdown	10·6^b^	4·0^a,b^	6·1^a^	10·6	8·9^a^	9·5^a^	8·7	17·1^a^	14·1^a^	8·8
Tea										
Pre-lockdown	20·1^b^	12·9^b^	15·1	20·4^b^	11·5^b^	14·6	28·0	9·3	16·2	14·8
Post-lockdown	19·5	14·9	16·4	22·6^b^	13·2^b^	16·5	30·4^b^	9·8^b^	17·2	16·5
Other beverages										
Pre-lockdown	18·2	22·7^a^	21·3^a^	18·8	16·4^a^	17·3^a^	16·0	9·3^a^	11·8^a^	18·1
Post-lockdown	23·2	25·2^a^	24·6^a^	19·6	19·0^a^	19·3^a^	8·7	2·4^a^	4·7^a^	20·3*
Individual-level changes in drink consumption										
Sugar-sweetened beverages										
Never	66·1^b^	73·8^a,b^	71·9^a^	59·9^b^	63·5^a,b^	62·4^a^	66·2	70·5^a^	69·2^a^	65·2
Constant	15·9^b^	12·3^a,b^	13·2^a^	20·8^b^	17·0^a,b^	18·2^a^	19·1	10·2^a^	12·8^a^	16·6
Increased	7·1^b^	4·8^a,b^	5·4^a^	6·5^b^	6·4^a,b^	6·4^a^	5·9	9·1^a^	8·2^a^	6·2
Decreased	10·9^b^	9·1^a,b^	9·5^a^	12·8^b^	13·1^a,b^	13·0^a^	8·8	10·2^a^	9·8^a^	12·0
Coffee and caffeinated drink										
Never	94·5^b^	98·5^a,b^	97·6^a^	93·6^b^	95·9^a,b^	95·2^a^	95·6	95·2^a^	95·2^a^	95·8
Constant	1·0^b^	0·6^a,b^	0·6^a^	2·2^b^	1·3^a,b^	1·6^a^	2·9	3·0^a^	3·0^a^	1·4
Increased	2·9^b^	0·4^a,b^	1·0^a^	1·8^b^	1·3^a,b^	1·4^a^	0	1·2^a^	0·9^a^	1·3
Decreased	1·6^b^	0·5^a,b^	0·8^a^	2·4^b^	1·5^a,b^	1·8^a^	1·5	0·6^a^	0·9^a^	1·5
Tea										
Never	88·8^b^	94·8^b^	93·4	88·0^b^	93·8^b^	92·1	88·2^b^	95·8^b^	93·6	92·5
Constant	3·8^b^	2·0^b^	2·4	5·7^b^	1·9^b^	3·0	8·8^b^	0·6^b^	3·0	2·9
Increased	3·3^b^	1·5^b^	1·9	2·8^b^	2·0^b^	2·3	1·5^b^	1·8^b^	1·7	2·1
Decreased	4·1^b^	1·7^b^	2·3	3·5^b^	2·3^b^	2·6	1·5^b^	1·8^b^	1·7	2·5
Other beverages										
Never	89·2	90·8	90·4^a^	88·7^b^	91·2^b^	90·5^a^	94·1	97·0	96·1^a^	90·6
Constant	4·9	3·1	3·5^a^	4·6^b^	2·7^b^	3·3^a^	3·0	0	0·9^a^	3·3
Increased	3·5	2·8	3·0^a^	2·8^b^	2·9^b^	2·8^a^	0	0·6	0·4^a^	2·8
Decreased	2·4	3·3	3·1^a^	3·9^b^	3·2^b^	3·4^a^	2·9	2·4	2·6^a^	3·3

Values under a given variable were marked with the superscript a, if the difference across educational levels (high school students, undergraduate students and graduate students) within the overall population (total) or within a given sex (male, female) was significant (*P* < 0·05); marked with the superscript b, if the difference between sex within a given educational level was significant (*P* < 0·05); and marked by asterisks, if the difference before and after lockdown within a given educational level and sex was significant (*P < 0·05, **P < 0·01, ***P < 0·001).

In the analyses for individual-level changes in twelve major food groups and beverages, we observed that a larger percentage of participants stopped or reduced the intake of beverages (15·1 %) compared to those who started or increased the intake (9·1 %) (Table 5). More participants started or increased their intake of wheat products (8·3 %), other staple foods (8·6 %), fish (7·5 %), eggs (10·3 %), fresh vegetables (7·6 %), preserved vegetables (9·0 %), fresh fruit (10·3 %) and dairy product (10·1 %) compared to those who stopped or decreased their intake of those foods. However, more participants stopped or reduced their intake of rice (5·9 %), meat (8·4 %), poultry (9·5 %) and soyabean products (9·2 %) compared to those who started or increased their intake of those foods. Similar changes in the dietary intake were observed in different subgroups stratified by educational level and sex (Table 5).

**Table 5:** Individual-level changes in dietary patterns among participating youths after lockdown

	Percentage									
	High school students			Undergraduate students			Graduate students			All
Variables	Male (*n* 678)	Female (*n* 2146)	Total (*n* 2824)	Male (*n* 2106)	Female (*n* 4918)	Total (*n* 7024)	Male (*n* 68)	Female (*n* 166)	Total (*n* 234)	Grand total (*n* 10 082)
Beverages										
Never	52·5^b^	65·8^ab^	62·7^a^	47·5^b^	56·5^ab^	53·7^a^	55·8	65·1^a^	62·4^a^	56·4
Constant	23·6^b^	14·9^ab^	17·0^a^	25·3^b^	18·4^ab^	20·5^a^	20·6	10·8^a^	13·7^a^	19·4
Increased	2·9^b^	1·2^ab^	1·6^a^	2·6^b^	1·8^ab^	2·1^a^	4·4	2·5^a^	3·0^a^	1·9
Decreased	1·8^b^	1·2^ab^	1·3^a^	2·1^b^	1·5^ab^	1·7^a^	1·5	1·8^a^	1·7^a^	1·6
Started consuming	7·8^b^	6·0^ab^	6·4^a^	7·4^b^	7·5^ab^	7·4^a^	7·4	9·0^a^	8·5^a^	7·2
Stopped consuming	11·4^b^	10·9^ab^	11·0^a^	15·1^b^	14·3^ab^	14·6^a^	10·3	10·8^a^	10·7^a^	13·5
Rice										
Never	6·3^ab^	3·4^ab^	4·1^a^	10·6^ab^	6·1^ab^	7·4^a^	11·8^a^	9·0^a^	9·8^a^	6·6
Constant	89·2^ab^	90·4^ab^	90·1^a^	82·3^ab^	82·2^ab^	82·2^a^	70·5^a^	66·3^a^	67·5^a^	84·1
Increased	1·5^ab^	2·0^ab^	1·8^a^	1·7^ab^	3·9^ab^	3·2^a^	4·4^a^	5·4^a^	5·1^a^	2·9
Decreased	1·6^ab^	2·9^ab^	2·6^a^	3·1^ab^	4·8^ab^	4·3^a^	7·4^a^	10·2^a^	9·4^a^	3·9
Started eating	0·7^ab^	0·2^ab^	0·4^a^	0·7^ab^	0·5^ab^	0·7^a^	1·5^a^	0·7^a^	0·9^a^	0·5
Stopped eating	0·7^ab^	1·1^ab^	1·0^a^	1·6^ab^	2·5^ab^	2·2^a^	4·4^a^	8·5^a^	7·3^a^	2·0
Wheat products										
Never	15·6	13·6	14·1^a^	15·9^b^	13·3^b^	14·1^a^	25·0	15·7	18·4^a^	14·2
Constant	71·2	70·1	70·4^a^	71·3^b^	68·8^b^	69·6^a^	55·9	57·8	57·2^a^	69·5
Increased	4·3	4·8	4·7^a^	5·3^b^	5·4^b^	5·4^a^	10·3	9·6	9·8^a^	5·3
Decreased	2·4	2·8	2·7^a^	2·8^b^	3·3^b^	3·1^a^	1·5	3·1	2·6^a^	3·0
Started eating	2·2	3·0	2·8^a^	2·0^b^	3·5^b^	3·0^a^	2·9	6·6	5·6^a^	3·0
Stopped eating	4·3	5·7	5·3^a^	2·7^b^	5·7^b^	4·8^a^	4·4	7·2	6·4^a^	5·0
Other staple foods										
Never	40·3^b^	46·7^ab^	45·2^a^	40·2^b^	39·5^ab^	39·7^a^	42·6	33·1^a^	35·9^a^	41·2
Constant	48·7^b^	40·9^ab^	42·6^a^	49·7^b^	44·9^ab^	46·3^a^	44·1	39·8^a^	41·0^a^	45·2
Increased	2·7^b^	1·9^ab^	2·1^a^	2·1^b^	2·6^ab^	2·5^a^	2·9	6·6^a^	5·6^a^	2·4
Decreased	1·9^b^	1·2^ab^	1·4^a^	1·5^b^	1·2^ab^	1·3^a^	1·6	3·1^a^	2·6^a^	1·3
Started eating	3·2^b^	5·4^ab^	4·9^a^	4·1^b^	7·8^ab^	6·7^a^	4·4	7·8^a^	6·8^a^	6·2
Stopped eating	3·2^b^	3·9^ab^	3·8^a^	2·4^b^	4·0^ab^	3·5^a^	4·4	9·6^a^	8·1^a^	3·7
Meat										
Never	9·1^b^	10·9^ab^	10·5	10·8^b^	8·5^ab^	9·2	10·3	8·4^a^	9·0	9·5
Constant	80·7^b^	73·4^ab^	75·1	77·2^b^	74·4^ab^	75·2	70·6	72·9^a^	72·1	75·1
Increased	3·5^b^	4·1^ab^	4·0	3·8^b^	5·9^ab^	5·3	5·9	5·4^a^	5·6	4·9
Decreased	3·2^b^	5·5^ab^	4·9	4·2^b^	5·4^ab^	5·1	5·9	7·8^a^	7·3	5·1
Started eating	1·4^b^	2·1^ab^	2·0	1·3^b^	2·3^ab^	2·0	2·9	1·3^a^	1·7	2·1
Stopped eating	2·1^b^	4·0^ab^	3·5	2·7^b^	3·5^ab^	3·2	4·4	4·2^a^	4·3	3·3
Poultry										
Never	24·8^b^	33·8^ab^	31·6^a^	22·8^b^	24·6^ab^	24·0^a^	17·6	12·7^a^	14·1^a^	25·9
Constant	59·7^b^	50·2^ab^	52·5^a^	61·6^b^	56·5^ab^	58·0^a^	60·3	66·9^a^	65·0^a^	56·7
Increased	4·0^b^	1·6^ab^	2·2^a^	4·2^b^	2·9^ab^	3·3^a^	4·4	4·8^a^	4·7^a^	3·0
Decreased	2·5^b^	1·6^ab^	1·8^a^	2·2^b^	2·3^ab^	2·4^a^	5·9	6·0^a^	6·0^a^	2·3
Started eating	3·1^b^	5·1^ab^	4·6^a^	3·3^b^	5·9^ab^	5·1^a^	1·5	4·8^a^	3·8^a^	4·9
Stopped eating	5·9^b^	7·7^ab^	7·3^a^	5·9^b^	7·8^ab^	7·2^a^	10·3	4·8^a^	6·4^a^	7·2
Fish										
Never	60·1^b^	72·5^ab^	69·1^a^	55·6^b^	61·0^ab^	59·4^a^	42·6	38·6^a^	39·7^a^	61·8
Constant	28·5^b^	16·8^ab^	19·7^a^	33·2^b^	25·0^ab^	27·5^a^	45·6	36·7^a^	39·3^a^	25·5
Increased	0·9^b^	1·0^ab^	1·1^a^	1·8^b^	1·0^ab^	1·2^a^	3·0	2·4^a^	2·6^a^	1·2
Decreased	1·0^b^	0·4^ab^	0·6^a^	1·0^b^	0·5^ab^	0·6^a^	0	1·8^a^	1·3^a^	0·6
Started eating	4·3^b^	4·9^ab^	4·9^a^	4·3^b^	7·9^ab^	6·8^a^	4·4	13·3^a^	10·7^a^	6·3
Stopped eating	5·2^b^	4·4^ab^	4·6^a^	4·1^b^	4·6^ab^	4·5^a^	4·4	7·2^a^	6·4^a^	4·6
Eggs										
Never	14·3^b^	17·1^ab^	16·5^a^	14·2^b^	11·5^ab^	12·3^a^	10·3	9·6^a^	9·8^a^	13·4
Constant	72·3^b^	65·8^ab^	67·2^a^	73·8^b^	70·1^ab^	71·2^a^	79·4	68·7^a^	71·8^a^	70·1
Increased	3·8^b^	4·1^ab^	4·0^a^	4·5^b^	6·9^ab^	6·2^a^	5·9	14·5^a^	12·0^a^	5·7
Decreased	2·8^b^	2·1^ab^	2·3^a^	2·8^b^	2·7^ab^	2·7^a^	1·5	4·2^a^	3·4^a^	2·6
Started eating	3·1^b^	5·7^ab^	5·1^a^	2·3^b^	5·4^ab^	4·5^a^	0	1·2^a^	0·9^a^	4·6
Stopped eating	3·7^b^	5·2^ab^	4·9^a^	2·4^b^	3·4^ab^	3·1^a^	2·9	1·8^a^	2·1^a^	3·6
Fresh vegetables										
Never	5·6^b^	3·4^ab^	3·9^a^	7·4^b^	2·8^ab^	4·2^a^	5·9	3·6^a^	4·3^a^	4·1
Constant	87·2^b^	86·0^ab^	86·4^a^	82·7^b^	83·2^ab^	83·1^a^	83·8	81·9^a^	82·5^a^	84·0
Increased	2·7^b^	5·8^ab^	5·0^a^	4·9^b^	8·0^ab^	7·1^a^	2·9	7·8^a^	6·4^a^	6·5
Decreased	2·9^b^	3·2^ab^	3·1^a^	2·9^b^	3·4^ab^	3·2^a^	4·4	3·6^a^	3·8^a^	3·2
Started eating	0·4^b^	0·6^ab^	0·5^a^	0·8^b^	1·6^ab^	1·3^a^	0	1·8^a^	1·3^a^	1·1
Stopped eating	1·2^b^	1·0^ab^	1·1^a^	1·3^b^	1·0^ab^	1·1^a^	3·0	1·3^a^	1·7^a^	1·1
Preserved vegetables										
Never	36·1^ab^	48·7^b^	45·7^a^	37·1^ab^	47·1^b^	43·9^a^	52·9^a^	52·4	52·6^a^	44·7
Constant	52·2^ab^	36·8^b^	40·5^a^	48·5^ab^	36·8^b^	40·4^a^	25·0^a^	28·9	27·8^a^	40·1
Increased	1·6^ab^	1·9^b^	1·8^a^	3·1^ab^	2·2^b^	2·5^a^	1·5^a^	1·8	1·7^a^	2·3
Decreased	1·1^ab^	0·7^b^	0·8^a^	1·6^ab^	1·2^b^	1·3^a^	2·9^a^	0·7	1·3^a^	1·2
Started eating	3·2^ab^	6·6^b^	5·8^a^	5·2^ab^	7·7^b^	7·0^a^	11·8^a^	11·4	11·5^a^	6·7
Stopped eating	5·8^ab^	5·3^b^	5·4^a^	4·5^ab^	5·0^b^	4·9^a^	5·9^a^	4·8	5·1^a^	5·0
Fresh fruit										
Never	11·1^b^	6·7^ab^	7·7^a^	12·0^b^	5·7^ab^	7·6^a^	10·3	4·8^a^	6·4^a^	7·6
Constant	78·4^b^	76·8^ab^	77·2^a^	73·3^b^	74·7^ab^	74·3^a^	75·0	68·1^a^	70·1^a^	75·0
Increased	3·1^b^	5·8^ab^	5·2^a^	5·7^b^	9·5^ab^	8·4^a^	5·9	14·5^a^	12·0^a^	7·6
Decreased	3·4^b^	4·5^ab^	4·2^a^	3·1^b^	4·4^ab^	4·0^a^	4·4	7·8^a^	6·8^a^	4·1
Started eating	1·5^b^	2·6^ab^	2·3^a^	3·0^b^	2·9^ab^	2·8^a^	2·9	1·8^a^	2·1^a^	2·7
Stopped eating	2·5^b^	3·6^ab^	3·4^a^	2·9^b^	2·8^ab^	2·9^a^	1·5	3·0^a^	2·6^a^	3·0
Soyabean products										
Never	39·7^ab^	50·1^ab^	47·6^a^	33·3^ab^	36·7^ab^	35·7^a^	27·9^a^	23·5^a^	24·8^a^	38·8
Constant	49·3^ab^	35·1^ab^	38·5^a^	52·6^ab^	45·8^ab^	47·9^a^	58·8^a^	51·2^a^	53·4^a^	45·3
Increased	0·9^ab^	1·6^ab^	1·5^a^	3·7^ab^	2·8^ab^	3·1^a^	4·4^a^	7·2^a^	6·4^a^	2·7
Decreased	1·6^ab^	1·8^ab^	1·8^a^	2·7^ab^	2·6^ab^	2·5^a^	1·5^a^	4·3^a^	3·4^a^	2·4
Started eating	2·9^ab^	4·8^ab^	4·3^a^	2·8^ab^	4·2^ab^	3·8^a^	1·5^a^	6·0^a^	4·7^a^	4·0
Stopped eating	5·6^ab^	6·6^ab^	6·3^a^	4·9^ab^	7·9^ab^	7·0^a^	5·9^a^	7·8^a^	7·3^a^	6·8
Dairy products										
Never	19·6^b^	23·7^ab^	22·7^a^	17·0^b^	15·0^ab^	15·6^a^	14·7	12·7^a^	13·2^a^	17·5
Constant	65·2^b^	55·8^ab^	58·1^a^	68·9^b^	64·3^ab^	65·7^a^	67·6	63·3^a^	64·5^a^	63·6
Increased	3·8^b^	4·5^ab^	4·3^a^	4·6^b^	6·8^ab^	6·1^a^	5·9	7·2^a^	6·8^a^	5·6
Decreased	2·6^b^	2·4^ab^	2·5^a^	3·1^b^	3·6^ab^	3·5^a^	3·0	5·4^a^	4·8^a^	3·2
Started eating	3·5^b^	6·1^ab^	5·5^a^	3·3^b^	4·4^ab^	4·0^a^	4·4	6·6^a^	6·0^a^	4·5
Stopped eating	5·3^b^	7·5^ab^	6·9^a^	3·1^b^	5·9^ab^	5·1^a^	4·4	4·8^a^	4·7^a^	5·6

Values under a given variable were marked with the superscript a, if the difference across educational levels (high school students, undergraduate students and graduate students) within the overall population (total) or within a given sex (male, female) was significant (*P* < 0·05); and marked with the superscript b, if the difference between sex within a given educational level was significant (*P* < 0·05).

## Discussion

We compared the patterns of a variety of food and beverage consumption before and after lockdown among 10 082 youths of three educational levels in China. A previous study conducted in Italy had verified the impact of lockdown on dietary patterns^(20)^. Our study further implied that such changes may persist even after lockdown. Significant differences of food and beverage consumption between the two time points were observed, including less frequent consumption of rice and SSB, and more frequent consumption of wheat products, other staple foods, fish, eggs, fresh vegetables, preserved vegetables, fresh fruit, dairy products and other beverages. Heterogeneities existed between sex and across educational levels to different extents. The structures of, rather than the food items under, the main dietary patterns have changed after lockdown: the pattern of dairy products remained the most consumed and the pattern of wheat products became the second dominant in food consumption. There might be several reasons for the changed consumption of food above-mentioned after lockdown. First, as a result of the pandemic, food choices in local groceries have been more limited than usual, which may decrease consumption of other foods than the types above. Second, staying at home for the pandemic, especially during the Chinese Spring Festival, may have left individuals with lots of frozen, canned and homemade food to be consumed for a longer period of time. The increases in the proportion of wheat products and preserved vegetables implies that people may consume convenient food more frequently after lockdown and stock up more food that can last longer (e.g. bread, canned food).

Diet is one of the foremost contributors to health^(21,22)^. These changes are of concern for potential long-term effect on youths’ health outcomes, especially when the lockdown lifted might last for a longer time since the pandemic seems far away from the end with probable outbreak recurrence^(12)^. For instance, we observed more intake of preserved vegetables. According to a systematic review, adulthood consumption of preserved vegetables (even in a low consumption level) are generally associated with a higher risk of nasopharyngeal carcinoma^(23)^, so the excessive intake of preserved vegetable is not recommended among youths. In addition to the negative changes, some positive changes after lockdown were also observed in our survey, such as an increase in fresh vegetable intakes, and a decrease in SSB consumption, which were all beneficial to health. However, it is difficult in our study to determine whether these changes are beneficial or harmful to youths’ health for not measuring personal nutritious intake precisely for large population surveys in a short time. However, the differences between the two time points shown in our epidemiological survey suggested the objective existence of changes in the dietary patterns, which implies the need to further examine these changes at the nutrient level. Thus, more studies relied on a mix of epidemiologic and interdisciplinary methods should be invited to China. For instance, the utilisation of 24-hour diet recall and diet records in a representative small population sample, which had been carried out in Australia to estimate the impact of lockdown on energy intake among youths^(24)^, or measuring the population’s intake of various nutrients at the molecular level.

Our findings of the potential long-term changed dietary patterns among youths provide with information that might help public health authorities reshape future policies on their nutritional recommendations, in preparation for future pandemics^(25)^. Interventions and education targeted healthy eating habits from schools and communities should be of consideration. Besides, more concerns and researches about this issue among youths should be invited to other counties. A previous study examined the dietary patterns of 509 youths through the method of 24-h diet recall^(24)^. However, there was limited evidence about the changes of dietary patterns after lockdown for youths. In view of a different context from China, different levels of lockdown measures as the response to the COVID-19 emergency and different provision of foods, we speculated different pictures of dietary patterns among youths in different countries^(5,25)^. Western countries need more evidence about the change after lockdown, which might be helpful for the early dietary provision and diet education.

The study should be treated with caution because of several limitations. First, we did not measure the amount of consumption for each types of food. Second, all participants were asked to report what happened 3–4 months ago, including a variety of foods, which may be subject to recall bias. However, their answers could still reflect the perceived changes of dietary patterns which are usually highly correlated with their actual changes of dietary patterns. Third, participants with different interpretations of the same question might affect the comparability of results. However, we have specifically listed examples of food at the end of each food category, which might help them understand the questions better. Fourth, to avoid the impact of overlength on the quality and even the completeness of responses, some questions that might help better explain the current findings were not asked in the survey. Fifth, the changes in diet patterns might take some time to change back to pre-pandemic status, as our survey was done right after the lockdown was lifted, we were not able to capture that change. Lastly, the participating youths were students and not from all cities in China, so our findings may not be generalised to the overall population in China without caution.

Despite all aforementioned limitations, there are still some important public health implications. For instance, our study provided a comprehensive picture of changes of dietary patterns among youths in China for the first time, and the survey tool used in the study could be also employed in other countries. Besides, this study revealed that the unhealthy eating patterns which underlie the changed consumption may last for a prolonged time period, which could contribute to more severe overweight/obesity issues and even more chronic disease burdens at later stages^(26,27)^. Thus, our findings imply some practical significance that interventions and health education from the public health sector are necessary after lockdown to improve the dietary quality among youths in order to avoid the development of unhealthy dietary behaviours.

In conclusion, this large-scale nationwide survey provided a basis of the changes of dietary patterns among youths after lockdown in China. The information would be useful for multiple stakeholders to learn the *status quo* of dietary patterns among the Chinese youths and to consider potential effects. For the Chinese government and health authorities, including policymakers and health professionals, when making future decisions regarding when and how to loosen restrictions, this issue of how to maintain healthy dietary patterns among youths cannot be ignored. Higher-quality experimental and intervention studies are appealed to set-up to thoroughly research on the impact of lockdown lifted on the dietary patterns.

## Supplementary material

For supplementary material accompanying this paper visit http://dx.doi.org/10.1017/S1368980020005170. click here to view supplementary material
