# Supplementary material for: Impacts of lockdown on dietary patterns among youths in China: the COVID-19 Impact on Lifestyle Change Survey
Source: Public Health Nutr. 2021 May 17:1–12. doi: 10.1017/S1368980020005170 (PMC8144820; doi:10.1017/S1368980020005170)
Supplement: Supplementary file 1 [file S1368980020005170sup001.docx]

**Table S1.** The weekly frequency of major food intake among participating youths before and after lockdown

| Variables | Percentage | | | | | | | | | |
| --- | --- | --- | --- | --- | --- | --- | --- | --- | --- | --- |
|  | High school students | | | Undergraduate students | | | Graduate students | | | All |
|  | Male  (n=678) | Female  (n=2,146) | Total  (n=2,824) | Male  (n=2,106) | Female  (n=4,918) | Total  (n=7,024) | Male  (n=68) | Female  (n=166) | Total  (n=234) | Grand total (n=10,082) |
| **Rice** |  |  |  |  |  |  |  |  |  |  |
| ***Pre-lockdown*** |  |  |  |  |  |  |  |  |  |  |
| None | 7.1 ^a^ | 3.6 ^a^ | 4.5 ^a^ | 11.3 ^a^ | 6.7 ^a^ | 8.0 ^a^ | 13.2 ^a^ | 9.6 ^a^ | 10.7 ^a^ | 7.1 |
| 1-3 | 2.8 ^a^ | 4.2 ^a^ | 3.9 ^a^ | 8.4 ^a^ | 11.5 ^a^ | 10.6 ^a^ | 14.7 ^a^ | 11.4 ^a^ | 12.4 ^a^ | 8.8 |
| 4-6 | 3.2 ^a^ | 3.4 ^a^ | 3.4 ^a^ | 6.3 ^a^ | 7.3 ^a^ | 7.0 ^a^ | 11.8 ^a^ | 16.3 ^a^ | 15.0 ^a^ | 6.2 |
| 7 | 86.9 ^a^ | 88.8 ^a^ | 88.2 ^a^ | 74.0 ^a^ | 74.5 ^a^ | 74.4 ^a^ | 60.3 ^a^ | 62.7 ^a^ | 61.9 ^a^ | 77.9 |
| ***Post-lockdown*** |  |  |  |  |  |  |  |  |  |  |
| None | 6.9 ^a^ | 4.5 ^a***^ | 5.1 ^a***^ | 12.2 ^a***^ | 8.6 ^a***^ | 9.7 ^a***^ | 16.2 ^a^ | 17.5 ^a***^ | 17.1 ^a***^ | 8.6^***^ |
| 1-3 | 3.5 ^a^ | 4.4 ^a***^ | 4.2 ^a***^ | 9.0 ^a***^ | 11.3 ^a***^ | 10.6 ^a***^ | 16.2 ^a^ | 13.9 ^a***^ | 14.5 ^a***^ | 8.9^***^ |
| 4-6 | 3.6 ^a^ | 4.2 ^a***^ | 4.0 ^a***^ | 6.7 ^a***^ | 7.3 ^a***^ | 7.1 ^a***^ | 8.8 ^a^ | 14.5 ^a***^ | 12.8 ^a***^ | 6.4^***^ |
| 7 | 86.0 ^a^ | 86.9 ^a***^ | 86.7 ^a***^ | 72.1 ^a***^ | 72.8 ^a***^ | 72.6 ^a***^ | 58.8 ^a^ | 54.1 ^a***^ | 55.6 ^a***^ | 76.1^***^ |
| **Wheat products** |  |  |  |  |  |  |  |  |  |  |
| ***Pre-lockdown*** |  |  |  |  |  |  |  |  |  |  |
| None | 17.8 ^b^ | 16.6 ^b^ | 16.9 ^a^ | 17.9 ^b^ | 16.8 ^b^ | 17.1 ^a^ | 27.9 | 22.3 | 23.9 ^a^ | 17.2 |
| 1-3 | 45.7 ^b^ | 55.6 ^b^ | 53.4 ^a^ | 44.7 ^b^ | 55.0 ^b^ | 51.9 ^a^ | 35.3 | 56.0 | 50.0 ^a^ | 52.2 |
| 4-6 | 12.0 ^b^ | 12.3 ^b^ | 12.2 ^a^ | 15.0 ^b^ | 12.3 ^b^ | 13.1 ^a^ | 14.7 | 10.3 | 11.6 ^a^ | 12.9 |
| 7 | 24.5 ^b^ | 15.5 ^b^ | 17.5 ^a^ | 22.4 ^b^ | 15.9 ^b^ | 17.9 ^a^ | 22.1 | 11.4 | 14.5 ^a^ | 17.7 |
| ***Post-lockdown*** |  |  |  |  |  |  |  |  |  |  |
| None | 19.9 ^b^ | 19.2 ^b^ | 19.4 | 18.6 ^b**^ | 19.1 ^b*^ | 18.9^***^ | 29.4 | 22.9^*^ | 24.8^**^ | 19.2^***^ |
| 1-3 | 41.3 ^b^ | 51.2 ^b^ | 48.8 | 41.5 ^b**^ | 50.4 ^b*^ | 47.8^***^ | 30.9 | 46.4^*^ | 41.9^**^ | 47.9^***^ |
| 4-6 | 13.6 ^b^ | 12.9 ^b^ | 13.0 | 15.8 ^b**^ | 13.0 ^b*^ | 13.9^***^ | 8.8 | 12.0^*^ | 11.1^**^ | 13.6^***^ |
| 7 | 25.2 ^b^ | 16.7 ^b^ | 18.8 | 24.1 ^b**^ | 17.5 ^b*^ | 19.4^***^ | 30.9 | 18.7^*^ | 22.2^**^ | 19.3^***^ |
| **Other staple foods** |  |  |  |  |  |  |  |  |  |  |
| ***Pre-lockdown*** |  |  |  |  |  |  |  |  |  |  |
| None | 43.5 ^b^ | 52.1 ^a, b^ | 50.0 ^a^ | 44.3 ^b^ | 47.3 ^a, b^ | 46.4 ^a^ | 47.0 | 41.0 ^a^ | 42.7 ^a^ | 47.4 |
| 1-3 | 35.4 ^b^ | 35.3 ^a, b^ | 35.3 ^a^ | 33.4 ^b^ | 38.3 ^a, b^ | 36.8 ^a^ | 30.9 | 44.0 ^a^ | 40.2 ^a^ | 36.5 |
| 4-6 | 7.4 ^b^ | 4.4 ^a, b^ | 5.1 ^a^ | 8.2 ^b^ | 5.8 ^a, b^ | 6.5 ^a^ | 11.8 | 7.2 ^a^ | 8.5 ^a^ | 6.1 |
| 7 | 13.7 ^b^ | 8.2 ^a, b^ | 9.6 ^a^ | 14.1 ^b^ | 8.6 ^a, b^ | 10.3 ^a^ | 10.3 | 7.8 ^a^ | 8.6 ^a^ | 10.0 |
| ***Post-lockdown*** |  |  |  |  |  |  |  |  |  |  |
| None | 43.5 ^b^ | 50.7 ^a, b***^ | 48.9 ^a***^ | 42.6 ^b**^ | 43.4 ^a, b***^ | 43.2 ^a***^ | 47.0 | 42.8 ^a^ | 44.0 ^a^ | 44.8^***^ |
| 1-3 | 35.0 ^b^ | 35.2 ^a, b***^ | 35.2 ^a***^ | 34.1 ^b**^ | 39.6 ^a, b***^ | 37.9 ^a***^ | 32.4 | 37.3 ^a^ | 35.9 ^a^ | 37.1^***^ |
| 4-6 | 7.4 ^b^ | 5.3 ^a, b***^ | 5.8 ^a***^ | 8.9 ^b**^ | 7.2 ^a, b***^ | 7.7 ^a***^ | 10.3 | 7.9 ^a^ | 8.6 ^a^ | 7.2^***^ |
| 7 | 14.1 ^b^ | 8.8 ^a, b***^ | 10.1 ^a***^ | 14.4 ^b**^ | 9.8 ^a, b***^ | 11.2 ^a***^ | 10.3 | 12.0 ^a^ | 11.5 ^a^ | 10.9^***^ |
| **Meat** |  |  |  |  |  |  |  |  |  |  |
| ***Pre-lockdown*** |  |  |  |  |  |  |  |  |  |  |
| None | 10.5 ^b^ | 13.0 ^a, b^ | 12.5 ^a^ | 12.1 ^b^ | 10.8 ^a, b^ | 11.2 ^a^ | 13.2 | 9.6 ^a^ | 10.7 ^a^ | 11.6 |
| 1-3 | 38.5 ^b^ | 50.0 ^a, b^ | 47.2 ^a^ | 31.0 ^b^ | 43.5 ^a, b^ | 39.8 ^a^ | 33.8 | 33.7 ^a^ | 33.8 ^a^ | 41.7 |
| 4-6 | 18.0 ^b^ | 18.8 ^a, b^ | 18.6 ^a^ | 23.1 ^b^ | 19.1 ^a, b^ | 20.3 ^a^ | 13.3 | 16.9 ^a^ | 15.8 ^a^ | 19.7 |
| 7 | 33.0 ^b^ | 18.2 ^a, b^ | 21.7 ^a^ | 33.8 ^b^ | 26.6 ^a, b^ | 28.7 ^a^ | 39.7 | 39.8 ^a^ | 39.7 ^a^ | 27.0 |
| ***Post-lockdown*** |  |  |  |  |  |  |  |  |  |  |
| None | 11.2 ^b^ | 14.9 ^a, b**^ | 14.0 ^a**^ | 13.5 ^b^ | 12.0 ^a, b^ | 12.4 ^a^ | 14.7 | 12.7 ^a^ | 13.2 ^a, b^ | 12.9^**^ |
| 1-3 | 37.2 ^b^ | 49.2 ^a, b**^ | 46.3 ^a**^ | 29.7 ^b^ | 42.0 ^a, b^ | 38.3 ^a^ | 33.8 | 30.7 ^a^ | 31.6 ^a, b^ | 40.4^**^ |
| 4-6 | 19.5 ^b^ | 17.8 ^a, b**^ | 18.2 ^a**^ | 23.4 ^b^ | 19.4 ^a, b^ | 20.6 ^a^ | 13.3 | 20.5 ^a^ | 18.4 ^a, b^ | 19.9^**^ |
| 7 | 32.1 ^b^ | 18.1 ^a, b**^ | 21.5 ^a**^ | 33.4 ^b^ | 26.6 ^a, b^ | 28.7 ^a^ | 38.2 | 36.1 ^a^ | 36.8 ^a, b^ | 26.8^**^ |
| **Poultry** |  |  |  |  |  |  |  |  |  |  |
| ***Pre-lockdown*** |  |  |  |  |  |  |  |  |  |  |
| None | 27.9 ^a, b^ | 38.9 ^a, b^ | 36.3 ^a^ | 26.1 ^a, b^ | 30.4 ^a, b^ | 29.1 ^a^ | 19.1 ^a^ | 17.5 ^a^ | 17.9 ^a^ | 30.9 |
| 1-3 | 59.0 ^a, b^ | 53.1 ^a, b^ | 54.5 ^a^ | 55.3 ^a, b^ | 57.6 ^a, b^ | 57.0 ^a^ | 52.9 ^a^ | 63.3 ^a^ | 60.3 ^a^ | 56.3 |
| 4-6 | 7.1 ^a, b^ | 4.9 ^a, b^ | 5.4 ^a^ | 10.4 ^a, b^ | 7.4 ^a, b^ | 8.3 ^a^ | 17.6 ^a^ | 9.0 ^a^ | 11.5 ^a^ | 7.6 |
| 7 | 6.0 ^a, b^ | 3.1 ^a, b^ | 3.8 ^a^ | 8.2 ^a, b^ | 4.6 ^a, b^ | 5.6 ^a^ | 10.4 ^a^ | 10.2 ^a^ | 10.3 ^a^ | 5.2 |
| ***Post-lockdown*** |  |  |  |  |  |  |  |  |  |  |
| None | 30.7 ^b^ | 41.5 ^a, b**^ | 38.9 ^a*^ | 28.7 ^b^ | 32.4 ^a, b^ | 31.3 ^a^ | 27.9 | 17.5 ^a^ | 20.5 ^a^ | 33.2^*^ |
| 1-3 | 54.4 ^b^ | 50.5 ^a, b**^ | 51.4 ^a*^ | 51.4 ^b^ | 54.8 ^a, b^ | 53.7 ^a^ | 47.1 | 61.4 ^a^ | 57.3 ^a^ | 53.2^*^ |
| 4-6 | 8.7 ^b^ | 4.8 ^a, b**^ | 5.8 ^a*^ | 11.0 ^b^ | 8.0 ^a, b^ | 8.9 ^a^ | 13.2 | 12.7 ^a^ | 12.8 ^a^ | 8.1^*^ |
| 7 | 6.2 ^b^ | 3.2 ^a, b**^ | 3.9 ^a*^ | 8.9 ^b^ | 4.8 ^a, b^ | 6.1 ^a^ | 11.8 | 8.4 ^a^ | 9.4 ^a^ | 5.5^*^ |
| **Fish** |  |  |  |  |  |  |  |  |  |  |
| ***Pre-lockdown*** |  |  |  |  |  |  |  |  |  |  |
| None | 64.5 ^a, b^ | 77.4 ^a, b^ | 74.3 ^a^ | 59.9 ^a, b^ | 68.8 ^a, b^ | 66.1 ^a^ | 47.0 ^a^ | 51.8 ^a^ | 50.4 ^a^ | 68.1 |
| 1-3 | 29.8 ^a, b^ | 20.1 ^a, b^ | 22.5 ^a^ | 31.7 ^a, b^ | 27.6 ^a, b^ | 28.8 ^a^ | 45.6 ^a^ | 39.8 ^a^ | 41.5 ^a^ | 27.3 |
| 4-6 | 3.2 ^a, b^ | 1.6 ^a, b^ | 2.0 ^a^ | 4.8 ^a, b^ | 2.2 ^a, b^ | 3.0 ^a^ | 7.4 ^a^ | 6.6 ^a^ | 6.8 ^a^ | 2.8 |
| 7 | 2.5 ^a, b^ | 0.9 ^a, b^ | 1.2 ^a^ | 3.6 ^a, b^ | 1.4 ^a, b^ | 2.1 ^a^ | 0 ^a^ | 1.8 ^a^ | 1.3 ^a^ | 1.8 |
| ***Post-lockdown*** |  |  |  |  |  |  |  |  |  |  |
| None | 65.3 ^a, b^ | 76.9 ^a, b^ | 74.1 ^a^ | 59.7 ^a, b^ | 65.6 ^a, b***^ | 63.8 ^a***^ | 47.1 ^a^ | 45.8 ^a^ | 46.2 ^a*^ | 66.3^***^ |
| 1-3 | 28.6 ^a, b^ | 20.2 ^a, b^ | 22.2 ^a^ | 31.0 ^a, b^ | 29.9 ^a, b***^ | 30.2 ^a***^ | 42.6 ^a^ | 44.0 ^a^ | 43.6 a^*^ | 28.3^***^ |
| 4-6 | 3.1 ^a, b^ | 1.6 ^a, b^ | 2.0 ^a^ | 4.9 ^a, b^ | 2.7 ^a, b***^ | 3.4 ^a***^ | 10.3 ^a^ | 7.8 ^a^ | 8.5 ^a*^ | 3.1^***^ |
| 7 | 3.0 ^a, b^ | 1.3 ^a, b^ | 1.7 ^a^ | 4.4 ^a, b^ | 1.8 ^a, b***^ | 2.6 ^a***^ | 0 ^a^ | 2.4 ^a^ | 1 .7 ^a*^ | 2.3^***^ |
| **Eggs** |  |  |  |  |  |  |  |  |  |  |
| ***Pre-lockdown*** |  |  |  |  |  |  |  |  |  |  |
| None | 17.4 ^a, b^ | 22.8 ^a, b^ | 21.5 ^a^ | 16.5 ^a, b^ | 16.9 ^a, b^ | 16.8 ^a^ | 10.3 ^a^ | 10.8 ^a^ | 10.7 ^a^ | 18.0 |
| 1-3 | 52.8 ^a, b^ | 58.7 ^a, b^ | 57.3 ^a^ | 44.5 ^a, b^ | 48.4 ^a, b^ | 47.2 ^a^ | 44.1 ^a^ | 36.7 ^a^ | 38.9 ^a^ | 49.8 |
| 4-6 | 15.0 ^a, b^ | 10.2 ^a, b^ | 11.3 ^a^ | 18.3 ^a, b^ | 15.5 ^a, b^ | 16.3 ^a^ | 16.2 ^a^ | 13.9 ^a^ | 14.5 ^a^ | 14.9 |
| 7 | 14.8 ^a, b^ | 8.3 ^a, b^ | 9.9 ^a^ | 20.7 ^a, b^ | 19.2 ^a, b^ | 19.7 ^a^ | 29.4 ^a^ | 38.6 ^a^ | 35.9 ^a^ | 17.3 |
| ***Post-lockdown*** |  |  |  |  |  |  |  |  |  |  |
| None | 18.0 ^a, b^ | 22.4 ^a, b***^ | 21.3 ^a***^ | 16.6 ^a, b***^ | 14.8 ^a, b***^ | 15.4 ^a***^ | 13.2 ^a^ | 11.4 ^a**^ | 12.0 ^a***^ | 17^***^ |
| 1-3 | 50.0 ^a, b^ | 56.3 ^a, b***^ | 54.9 ^a***^ | 41.9 ^a, b***^ | 45.4 ^a, b***^ | 44.3 ^a***^ | 36.8 ^a^ | 25.9 ^a**^ | 29.1 ^a***^ | 46.9^***^ |
| 4-6 | 16.4 ^a, b^ | 12.3 ^a, b***^ | 13.2 ^a***^ | 19.3 ^a, b***^ | 17.6 ^a, b***^ | 18.1 ^a***^ | 19.1 ^a^ | 18.1 ^a**^ | 18.4 ^a***^ | 16.8^***^ |
| 7 | 15.6 ^a, b^ | 9.0 ^a,^ ^b***^ | 10.6 ^a***^ | 22.2 ^a, b***^ | 22.2 ^a, b***^ | 22.2 ^a***^ | 30.9 ^a^ | 44.6 ^a**^ | 40.5 ^a***^ | 19.3^***^ |
| **Fresh vegetables** |  |  |  |  |  |  |  |  |  |  |
| ***Pre-lockdown*** |  |  |  |  |  |  |  |  |  |  |
| None | 6.1 ^b^ | 3.9 ^a, b^ | 4.5 ^a^ | 8.2 ^b^ | 4.4 ^a, b^ | 5.5 ^a^ | 5.9 | 5.4 ^a^ | 5.6 ^a^ | 5.2 |
| 1-3 | 21.2 ^b^ | 16.4 ^a, b^ | 17.5 ^a^ | 19.7 ^b^ | 19.0 ^a, b^ | 19.2 ^a^ | 19.1 | 11.4 ^a^ | 13.7 ^a^ | 18.6 |
| 4-6 | 21.1 ^b^ | 17.6 ^a, b^ | 18.4 ^a^ | 20.7 ^b^ | 19.3 ^a, b^ | 19.7 ^a^ | 19.1 | 14.5 ^a^ | 15.8 ^a^ | 19.3 |
| 7 | 51.6 ^b^ | 62.1 ^a, b^ | 59.6 ^a^ | 51.4 ^b^ | 57.3 ^a, b^ | 55.6 ^a^ | 55.9 | 68.7 ^a^ | 64.9 ^a^ | 56.9 |
| ***Post-lockdown*** |  |  |  |  |  |  |  |  |  |  |
| None | 6.8 ^b^ | 4.4 ^a, b**^ | 4.9 ^a*^ | 8.6 ^b*^ | 3.8 ^a, b***^ | 5.2 ^a***^ | 8.8 ^b^ | 4.8 ^a, b^ | 6.0 ^a^ | 5.2^***^ |
| 1-3 | 20.4 ^b^ | 15.2 ^a, b**^ | 16.5 ^a*^ | 17.9 ^b*^ | 16.1 ^a, b***^ | 16.7 ^a***^ | 22.1 ^b^ | 9.0 ^a, b^ | 12.8 ^a^ | 16.5^***^ |
| 4-6 | 21.3 ^b^ | 16.1 ^a, b**^ | 17.4 ^a*^ | 20.8 ^b*^ | 18.9 ^a, b***^ | 19.5 ^a***^ | 17.6 ^b^ | 13.9 ^a, b^ | 15.0 ^a^ | 18.8^***^ |
| 7 | 51.5 ^b^ | 64.3 ^a, b**^ | 61.2 ^a*^ | 52.7 ^b*^ | 61.2 ^a, b***^ | 58.6 ^a***^ | 51.5 ^b^ | 72.3 ^a, b^ | 66.2 ^a^ | 59.5^***^ |
| Preserved vegetables | |  |  |  |  |  |  |  |  |  |
| ***Pre-lockdown*** |  |  |  |  |  |  |  |  |  |  |
| None | 39.4 ^a, b^ | 55.3 ^b^ | 51.5 ^a^ | 42.2 ^a, b^ | 54.8 ^b^ | 51.0 ^a^ | 64.7 ^a^ | 63.9 | 64.1 ^a^ | 51.4 |
| 1-3 | 46.5 ^a, b^ | 37.6 ^b^ | 39.7 ^a^ | 42.0 ^a, b^ | 36.0 ^b^ | 37.8 ^a^ | 22.1 ^a^ | 27.7 | 26.1 ^a^ | 38.1 |
| 4-6 | 7.2 ^a, b^ | 3.6 ^b^ | 4.5 ^a^ | 7.3 ^a, b^ | 4.3 ^b^ | 5.2 ^a^ | 8.8 ^a^ | 3.6 | 5.1 ^a^ | 5.0 |
| 7 | 6.9 ^a, b^ | 3.5 ^b^ | 4.3 ^a^ | 8.5 ^a, b^ | 4.9 ^b^ | 6.0 ^a^ | 4.4 ^a^ | 4.8 | 4.7 ^a^ | 5.5 |
| ***Post-lockdown*** |  |  |  |  |  |  |  |  |  |  |
| None | 41.9 ^a, b^ | 54.0 ^a, b***^ | 51.1 ^a**^ | 41.5 ^a, b***^ | 52.1 ^a, b***^ | 48.9 ^a***^ | 58.8 ^a^ | 57.1 ^a*^ | 57.6 ^a*^ | 49.7^***^ |
| 1-3 | 43.2 ^a, b^ | 37. 5 ^a, b***^ | 38.8 ^a**^ | 40.7 ^a, b***^ | 36.6 ^a, b***^ | 37.9 ^a***^ | 29.4 ^a^ | 30.8 ^a*^ | 30.4 ^a*^ | 38^***^ |
| 4-6 | 7.4 ^a, b^ | 4.4 ^a, b***^ | 5.1 ^a**^ | 8.5 ^a, b***^ | 5.6 ^a, b***^ | 6.4 ^a***^ | 5.9 ^a^ | 4.8 ^a*^ | 5.1 ^a*^ | 6^***^ |
| 7 | 7.5 ^a, b^ | 4.1 ^a, b***^ | 5.0 ^a**^ | 9.3 ^a, b***^ | 5.7 ^a, b***^ | 6.8 ^a***^ | 5.9 ^a^ | 7.3 ^a*^ | 6.9 ^a*^ | 6.3^***^ |
| **Fresh fruit** |  |  |  |  |  |  |  |  |  |  |
| ***Pre-lockdown*** |  |  |  |  |  |  |  |  |  |  |
| None | 12.5 | 9.2 ^a^ | 10.0 ^a^ | 15.0 ^b^ | 8.5 ^a, b^ | 10.5 ^a^ | 13.3 ^b^ | 6.6 ^a, b^ | 8.5 ^a^ | 10.3 |
| 1-3 | 45.3 | 47.5 ^a^ | 47.0 ^a^ | 42.4 ^b^ | 41.3 ^a, b^ | 41.6 ^a^ | 38.2 ^b^ | 26.5 ^a, b^ | 29.9 ^a^ | 42.8 |
| 4-6 | 21.4 | 21.2 ^a^ | 21.2 ^a^ | 23.2 ^b^ | 22.0 ^a, b^ | 22.4 ^a^ | 29.4 ^b^ | 26.5 ^a, b^ | 27.4 ^a^ | 22.2 |
| 7 | 20.8 | 22.1 ^a^ | 21.8 ^a^ | 19.4 ^b^ | 28.2 ^a, b^ | 25.5 ^a^ | 19.1 ^b^ | 40.4 ^a, b^ | 34.2 ^a^ | 24.7 |
| ***Post-lockdown*** |  |  |  |  |  |  |  |  |  |  |
| None | 13.6 | 10.3 ^a^ | 11.1 ^a^ | 15.0 ^b***^ | 8.6 ^a, b***^ | 10.5 ^a***^ | 11.8 ^b^ | 7.8 ^a, b^ | 9.0 ^a^ | 10.6^***^ |
| 1-3 | 44.4 | 44.9 ^a^ | 44.8 ^a^ | 39.9 ^b***^ | 36.9 ^a, b***^ | 37.8 ^a***^ | 39.6 ^b^ | 19.9 ^a, b^ | 25.6 ^a^ | 39.5^***^ |
| 4-6 | 21.4 | 22.2 ^a^ | 22.0 ^a^ | 24.1 ^b***^ | 23.3 ^a, b***^ | 23.5 ^a***^ | 26.5 ^b^ | 28.3 ^a, b^ | 27.8 ^a^ | 23.2^***^ |
| 7 | 20.6 | 22.6 ^a^ | 22.1 ^a^ | 21.0 ^b***^ | 31.2 ^a, b***^ | 28.2 ^a***^ | 22.1 ^b^ | 44.0 ^a, b^ | 37.6 ^a^ | 26.7^***^ |
| **Soybean products** |  |  |  |  |  |  |  |  |  |  |
| ***Pre-lockdown*** |  |  |  |  |  |  |  |  |  |  |
| None | 42.6 ^a, b^ | 54.8 ^a, b^ | 51.9 ^a^ | 36.1 ^a, b^ | 41.1 ^a, b^ | 39.5 ^a^ | 29.4 ^a^ | 29.5 ^a^ | 29.5 ^a^ | 42.8 |
| 1-3 | 41.0 ^a, b^ | 35.5 ^a, b^ | 36.8 ^a^ | 44.8 ^a, b^ | 43.8 ^a, b^ | 44.2 ^a^ | 44.1 ^a^ | 45.2 ^a^ | 44.9 ^a^ | 42.1 |
| 4-6 | 7.7 ^a, b^ | 5.7 ^a, b^ | 6.2 ^a^ | 10.2 ^a, b^ | 7.4 ^a, b^ | 8.3 ^a^ | 14.7 ^a^ | 14.5 ^a^ | 14.5 ^a^ | 7.8 |
| 7 | 8.7 ^a, b^ | 4.0 ^a, b^ | 5.1 ^a^ | 8.9 ^a, b^ | 7.7 ^a, b^ | 8.0 ^a^ | 11.8 ^a^ | 10.8 ^a^ | 11.1 ^a^ | 7.3 |
| ***Post-lockdown*** |  |  |  |  |  |  |  |  |  |  |
| None | 45.3 ^a, b**^ | 56.7 ^a, b**^ | 53.9 ^a***^ | 38.3 ^a, b^ | 44.6 ^a, b***^ | 42.7 ^a***^ | 33.8 ^a^ | 31.3 ^a^ | 32.1 ^a^ | 45.5^***^ |
| 1-3 | 40.0 ^a, b**^ | 34.4 ^a, b**^ | 35.8 ^a***^ | 41.1 ^a, b^ | 41.1 ^a, b***^ | 41.1 ^a***^ | 36.8 ^a^ | 39.8 ^a^ | 38.9 ^a^ | 39.6^***^ |
| 4-6 | 6.9 ^a, b**^ | 5.3 ^a, b**^ | 5.7 ^a***^ | 12.1 ^a, b^ | 7.6 ^a, b***^ | 9.0 ^a***^ | 17.6 ^a^ | 17.5 ^a^ | 17.5 ^a^ | 8.3^***^ |
| 7 | 7.8 ^a, b**^ | 3.6 ^a, b**^ | 4.6 ^a***^ | 8.5 ^a, b^ | 6.7 ^a, b***^ | 7.2 ^a***^ | 11.8 ^a^ | 11.4 ^a^ | 11.5 ^a^ | 6.6^***^ |
| **Dairy products** |  |  |  |  |  |  |  |  |  |  |
| ***Pre-lockdown*** |  |  |  |  |  |  |  |  |  |  |
| None | 23.2 ^b^ | 29.8 ^a, b^ | 28.2 ^a^ | 20.3 | 19.4 ^a^ | 19.6 ^a^ | 19.1 | 19.3 ^a^ | 19.2 ^a^ | 22.0 |
| 1-3 | 42.2 ^b^ | 49.2 ^a, b^ | 47.6 ^a^ | 41.3 | 45.9 ^a^ | 44.4 ^a^ | 39.7 | 39.7 ^a^ | 39.7 ^a^ | 45.3 |
| 4-6 | 15.3 ^b^ | 11.0 ^a, b^ | 12.0 ^a^ | 16.8 | 15.8 ^a^ | 16.1 ^a^ | 13.2 | 16.3 ^a^ | 15.5 ^a^ | 14.9 |
| 7 | 19.3 ^b^ | 10.0 ^a, b^ | 12.2 ^a^ | 21.6 | 18.9 ^a^ | 19.9 ^a^ | 28.0 | 24.7 ^a^ | 25.6 ^a^ | 17.8 |
| ***Post-lockdown*** |  |  |  |  |  |  |  |  |  |  |
| None | 24.9 ^a, b^ | 31.1 ^a, b^ | 29.6 ^a^ | 20.2 ^a*^ | 20.9 ^a***^ | 20.7 ^a***^ | 19.1 ^a^ | 17.5 ^a^ | 17.9 ^a^ | 23.1^***^ |
| 1-3 | 40.0 ^a, b^ | 46.5 ^a, b^ | 44.9 ^a^ | 39.9 ^a*^ | 41.5 ^a***^ | 41.0 ^a***^ | 36.8 ^a^ | 42.1 ^a^ | 40.6 ^a^ | 42.1^***^ |
| 4-6 | 15.5 ^a, b^ | 11.7 ^a, b^ | 12.7 ^a^ | 17.5 ^a*^ | 16.8 ^a***^ | 17.0 ^a***^ | 14.7 ^a^ | 12.7 ^a^ | 13.3 ^a^ | 15.7^***^ |
| 7 | 19.6 ^a, b^ | 10.7 ^a, b^ | 12.8 ^a^ | 22.4 ^a*^ | 20.8 ^a***^ | 21.3 ^a***^ | 29.4 ^a^ | 27.7 ^a^ | 28.2 ^a^ | 19.1^***^ |

Values under a given variable were marked with the superscript *a*, if the difference across educational levels (High school students, Undergraduate students, Graduate students) within the overall population (Total) or within a given sex (Male, Female) was significant (*p*<0.05); marked with the superscript *b*, if the difference between sex within a given educational level was significant (*p*<0.05); and marked by asterisks, if the difference before and after lockdown within a given educational level and sex was significant (^*^*p*<0.05, ^**^*p*<0.01, ^***^*p*<0.001).
